# The significance of measuring monocyte tissue factor activity in patients with breast and colorectal cancer

**DOI:** 10.1038/sj.bjc.6690352

**Published:** 1999-04

**Authors:** B A Lwaleed, M Chisholm, J L Francis

**Affiliations:** University Department of Haematology, Level F(827), South Academic Block, Tremona Road, Southampton SO16 6YD, UK; Hemostasis and Thrombosis Research Unit, Walt Disney Memorial Cancer Institute at Florida Hospital, Altamonte Spring, FL 32701, USA

**Keywords:** monocyte tissue factor, coagulopathy, cancer

## Abstract

Monocytes express tissue factor (mTF) in several conditions including cancer where levels may be valuable in assessing tumour presence and progression. Using a two-stage kinetic chromogenic assay (KCA), mTF levels were measured in controls [normal subjects (*n* = 60) and patients undergoing hernia repair or cholecystectomy (*n* = 60)], in patients with benign and malignant disease of the breast (*n* = 83) and of the large bowel (*n* = 62). This was performed under fresh (resting) conditions and after incubation for 6 h without (unstimulated) and with (stimulated) *Escherichia coli* endotoxin. The malignant groups showed higher mTF levels than each of the three controls for resting (*P* < 0.05 breast, *P* < 0.05 colorectal) unstimulated (*P* < 0.05 breast, *P* < 0.05 colorectal) and stimulated cells (*P* < 0.001 breast, *P* < 0.01 colorectal). Similarly, the benign inflammatory groups had higher mTF levels than controls for resting (*P* < 0.05 colorectal), unstimulated (*P* < 0.05 colorectal) and stimulated cells (*P* < 0.01 breast, *P* < 0.01 colorectal). There was no significant difference between malignant and benign inflammatory groups in each organ. mTF levels showed an increase corresponding to that of histological tumour progression and were higher in non-surviving patients. In conclusion, mTF levels are raised in malignant and inflammatory disease compared to controls and patients with non-inflammatory conditions. Stimulated cells give better discrimination between the groups and may be of value in identifying high risk individuals. mTF levels showed an association with tumour grade or stage and the patients' survival time. © 1999 Cancer Research Campaign


*British Journal of Cancer* (1999) **80**(1/2), 279–285
© 1999 Cancer Research Campaign
Article no. bjoc.1998.0352

### The significance of measuring monocyte tissue factor activity in patients with breast and colorectal cancer

**BA Lwaleed[1], M Chisholm[1] and JL Francis[2]**

[1]University Department of Haematology, Level F(827), South Academic Block, Southampton University Hospitals, Tremona Road, Southampton SO16 6YD, UK; [2]Hemostasis and Thrombosis Research Unit, Walt Disney Memorial Cancer Institute at Florida Hospital, Altamonte Spring, FL 32701, USA

**Summary** Monocytes express tissue factor (mTF) in several conditions including cancer where levels may be valuable in assessing tumour presence and progression. Using a two-stage kinetic chromogenic assay (KCA), mTF levels were measured in controls [normal subjects ($n$ = 60) and patients undergoing hernia repair or cholecystectomy ($n$ = 60)], in patients with benign and malignant disease of the breast ($n$ = 83) and of the large bowel ($n$ = 62). This was performed under fresh (resting) conditions and after incubation for 6 h without (unstimulated) and with (stimulated) *Escherichia coli* endotoxin. The malignant groups showed higher mTF levels than each of the three controls for resting ($P$ < 0.05 breast, $P$ < 0.05 colorectal) unstimulated ($P$ < 0.05 breast, $P$ < 0.05 colorectal) and stimulated cells ($P$ < 0.001 breast, $P$ < 0.01 colorectal). Similarly, the benign inflammatory groups had higher mTF levels than controls for resting ($P$ < 0.05 colorectal), unstimulated ($P$ < 0.05 colorectal) and stimulated cells ($P$ < 0.01 breast, $P$ < 0.01 colorectal). There was no significant difference between malignant and benign inflammatory groups in each organ. mTF levels showed an increase corresponding to that of histological tumour progression and were higher in non-surviving patients. In conclusion, mTF levels are raised in malignant and inflammatory disease compared to controls and patients with non-inflammatory conditions. Stimulated cells give better discrimination between the groups and may be of value in identifying high risk individuals. mTF levels showed an association with tumour grade or stage and the patients' survival time.

**Keywords:** monocyte tissue factor; coagulopathy; cancer

Increased monocyte tissue factor (mTF) expression occurs in many inflammatory conditions, and its behaviour has been investigated in animal and human cancer. Circulating mononuclear cells from tumour-bearing animals have significantly more TF activity than those from control animals (Lorenzet et al, 1983). Patients with cancer have significantly higher levels compared with normal controls (Edwards et al, 1981; Morgan et al, 1988; Carvalho, 1995; Lwaleed et al, 1997) and cultured monocytes from cancer patients express increased amounts of TF compared to normal subjects (Edwards et al, 1981). Furthermore, mTF levels were linearly correlated with other markers of in vivo coagulation activation (as measured by elevation of fibrinogen peptide A level) and tumour burden (Edwards et al, 1981; Morgan et al, 1988; Auger and Mackie, 1987) suggesting that increased fibrin formation in vivo is at least partly dependent on mTF expression.

TF is a single-chain, integral plasma membrane glycoprotein with no intrinsic protease activity (Bach et al, 1981; Guha et al, 1986). TF serves as a receptor and essential co-factor for plasma factors VII and VIIa in rapid activation of factors X and IX (Nemerson and Bach, 1982). Thus TF is the principle physiological initiator of blood coagulation (Nemerson, 1992). Since monocytes are the only blood cells that are capable of synthesizing TF (Østerud and Bjorkild, 1982), the emergence of procoagulant activity (PCA) in response to stimulants such as lipopolysacchride (LPS) in whole blood level might be of clinical significance. Dasmahapatra et al (1987), using a whole blood re-calcification time showed an increased level of LPS-induced mTF in patients with various tumours and suggested that this could be used to distinguish, with some certainty, patients with malignant diseases from those with benign non-inflammatory diseases. Contrino et al (1996), demonstrated using both immunohistochemical technique and a novel probe for functional TF, that TF expression by endothelial cells is present only in the blood vessels of malignant breast lesions but not in the vessels of benign breast lesions. Thus both studies demonstrate an association between TF expression and the malignant phenotype, although the TF detected is on host cells either circulating or embedded in the tumour stroma. In contrast, Østerud and Due (1984) failed to find significant differences in mTF levels or indeed any other coagulation parameters between patients with malignant or benign tumours. Mellor et al (1989) reported higher whole blood PCA in breast and colorectal cancer patients compared to normal controls, but there was no distinction between malignant and benign surgical control groups. The latter studies, however, may not have paid enough attention to the benign groups since inflammatory conditions may cause similar rises in mTF levels (Edwards et al, 1987). Indeed Parmar et al (1990) showed a significant difference in whole bood PCA between cancer and non-cancer surgical groups, with cancer groups demonstrating higher levels.

While some have measured mTF PCA in whole blood (Dasmahapatra et al, 1987; Mellor et al, 1989; Parmar et al, 1990), others have employed laborious techniques to isolate monocytes (Muller et al, 1985) and have often measured the PCA in disrupted, rather than intact, cells (Østerud and Due, 1984). In the present study we employed a two-stage kinetic chromogenic assay (KCA) which measures mTF on intact cells isolated in a one-step

*Received 26 March 1998*
*Revised 28 September 1998*
*Accepted 20 October 1998*

*Correspondence to:* BA Lwaleed

**Table 1** Sample size of monocyte tissue factor activity, median and age range and sex for all groups studied for the neoplastic diseases

| Group | *n* | Median age (years) | Age range | Male (*n*) | Female (*n*) |
|---|---|---|---|---|---|
| Normals[a] | 60 | 35 | 19–77 | 26 | 34 |
| Cholecystectomy or hernia | 60 | 61 | 30–86 | 43 | 17 |
| Breast benign non-inflammatory | 21 | 42 | 20–74 | – | 21 |
| Breast benign inflammatory[b] | 4 | 57 | 42–76 | – | 4 |
| Breast cancer | 58 | 63 | 35–87 | – | 58 |
| Colorectal benign non-inflammatory | 31 | 66 | 39–92 | 16 | 15 |
| Colorectal benign inflammatory[c] | 13 | 60 | 44–89 | 7 | 6 |
| Colorectal cancer | 18 | 73 | 46–85 | 8 | 10 |

[a]Hospital staff; [b]mastitis and mammary duct ectasia; [c]ulcerative colitis and diverticulitis.

procedure using Leukoprep™ tubes which did not artifactually activate the cells (Carvalho, 1995). This assay is not significantly affected by age, gender or smoking habit. Using this assay we assessed mTF levels in patients with breast and colorectal cancer, and compared the results with normal controls, surgical controls and subjects with benign diseases. The effect of inflammatory conditions on mTF was assessed and mTF levels were correlated with histological tumour grading or staging and patient survival.

### MATERIALS AND METHODS

### Controls and patients

Ethical committee approval was obtained for the study from the Southampton and South West Hampshire Health Commission Joint Ethical Sub-Committee (submission number 239/93) and informed consent was sought from each patient. A total of 265 subjects were studied: Patients were admitted into the surgical wards of Southampton University Hospitals (see Table 1 for details). Blood samples were taken prior to operation. All patients had been clinically diagnosed and the histopathology reports were subsequently reviewed. Tumours were classified according to the World Health Organization classification for breast cancer and Dukes' categorization for colorectal cancer.

### Controls and patients mTF level was assayed using a two-stage KCA (Carvalho, 1995) which is described below. mTF activity was measured as baseline (resting cells), after 6 h incubation without endotoxin (unstimulated cells) and after 6 h incubation with endotoxin (10 μg/ml, Sigma Chemical Company, Pool, Dorset, UK) (stimulated cells) at 37°C and 5% carbon dioxide. TF on the surface of the monocytes forms a complex with factor VII and $Ca^{2+}$. The complex activates factor X to factor Xa which then acts on a specific chromogenic substrate resulting in the generation of a yellow colour that can then be measured spectrophotometrically.

### Cell preparation

Ten millilitres of venous blood was collected into an ethylene-diamine-tetra acetic acid (EDTA) tube. The blood samples were then transferred to Leukoprep™ tubes (Becton Dickinson, Oxford, UK) and centrifuged (within 2 h of sample collection) for 15 min at 1500–1800 ***g*** at room temperature. After centrifugation the plasma layer was discarded; the buffy coat layer, which contains the mononuclear cells as well as platelets, was transferred to a flat-bottomed polystyrene tube (Greiner Labortechnik, Germany). Cells were then washed three times using cooled sterilized Dulbecco's saline buffer (Sigma Chemical Company, Pool, Dorset, UK). The washed mononuclear cells were then resuspended in RPMI-1640 medium with L-glutamine (Gibco, UK) and adjusted to $2 \times 10^6$ cells $ml^{-1}$ and platelet to monocyte ratio of between 4:1 and 6:1.

### Assay procedure

A total of 100 μl of cell suspension were placed into duplicate wells in a 96-well flat-bottomed microtitre plate and cells were allowed to adhere for 10 min. The non-adherent cells and the pre-set ratio of contaminating platelets (see above) were then washed off using cold saline. To the adherent cells the following reagents were then added: 60 μl of the assay buffer (0.05 Tris, sodium chloride, pH 7.8), 20 μl of calcium chloride (0.025 M) and 15 μl (0.75 unit $ml^{-1}$) of a commercially available clotting factor concentrate (Prothromplex TIM4 30 U $ml^{-1}$, Immuno Ltd, Dunton Green, UK) as a source of factors VII and X. Control wells contained the above reagents, but with 15 μl of factor X buffer instead of prothromplex for background subtraction. The plate was then incubated at 37°C for 10 min. The reaction was then stopped with 100 μl of assay buffer containing 7.5 mM EDTA. A total of 40 μl of spectrozyme Xa was added and the rate of factor Xa generation, which is proportional to the amount of TF expressed by monocytes, was determined by measuring the increase in the absorbance of the free chromophore pNA generated in comparison to the original substrate. Absorbance values were automatically calculated by the Kinetic-Calc™ package based on the standard curve constructed from serial dilutions of recombinant relipidated TF (0.75–83.0 ng $ml^{-1}$) and converted to TF (ng $10^{-6}$ cells). The recombinant TF was obtained from the American Diagnostica Inc, Greenwich, CT, USA. Relipidation was performed in our laboratory according to the method of Carson and Konigsberg (1980). The standard curve is set out on each microplate for each set of measurements. Results were then multiplied by 5 and expressed as ng TF $10^{-6}$ mononuclear cells per well (each well contained $2 \times 10^6$ cells).

### Statistical analysis

Data were included in a database and analysed by the STATGRAPHICS™ statistical software system. Data were not normally distributed, and summary statistics were expressed as medians and interquartile ranges (IQR). Differences between two groups were assessed by the Mann–Whitney *U*-test. Differences in tumour grading were tested by Kruskal–Wallis one-way analysis by ranks.

© Cancer Research Campaign 1999

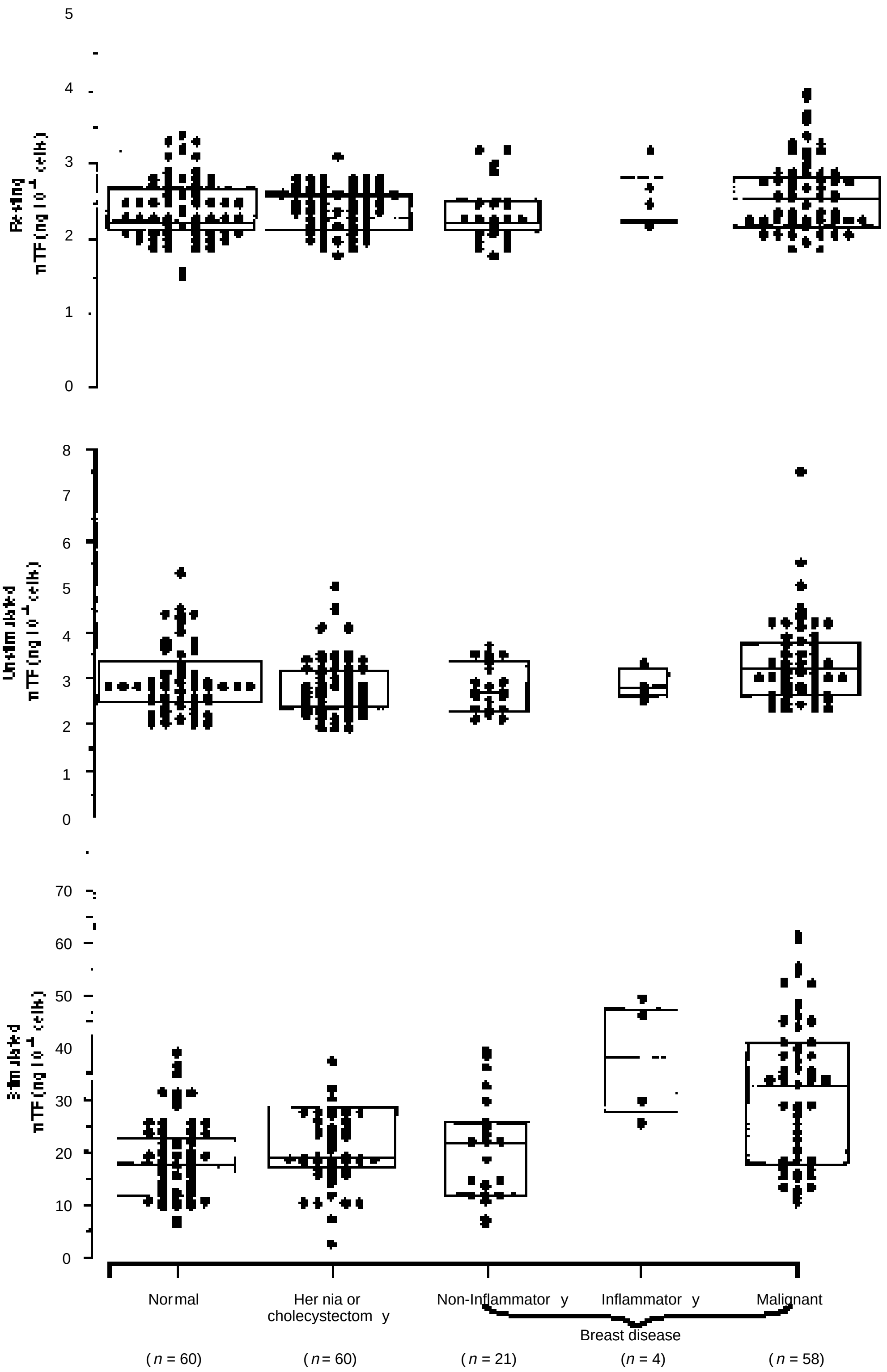

**Figure 1** Monocyte tissue factor activity (ng $10^{-6}$ cells) in normal controls, patients undergoing hernia repair or cholecystectomy, non-inflammatory and inflammatory benign disease, and malignant disease of the breast, for baseline (resting), after 6 h incubation without endotoxin (unstimulated cells) and after 6 h incubation with endotoxin (stimulated cells)

**Table 2** Differences in monocyte tissue factor between normal controls and various disease categories

| | Normal | Breast B-Non-Inf | Breast B-Inf | Breast cancer | Colorectal B-Non-Inf | Colorectal B-Inf | Colorectal cancer |
|---|---|---|---|---|---|---|---|
| Normal | – | NS | NS | < 0.01 | NS | < 0.05 | < 0.05 |
| | – | NS | NS | < 0.05 | NS | < 0.05 | < 0.05 |
| | – | NS | < 0.01 | < 0.001 | NS | < 0.01 | < 0.01 |
| Hernia and Chole | NS | NS | NS | < 0.01 | NS | < 0.05 | < 0.05 |
| | NS | NS | NS | < 0.01 | NS | < 0.05 | < 0.05 |
| | NS | NS | < 0.01 | < 0.001 | NS | < 0.01 | < 0.01 |
| Breast B-Non-Inf | NS | – | NS | < 0.05 | – | – | – |
| | NS | – | NS | < 0.05 | – | – | – |
| | NS | – | < 0.01 | < 0.001 | – | – | – |
| Colorectal B-Non-Inf | NS | – | – | – | – | < 0.05 | < 0.05 |
| | NS | – | – | – | – | < 0.01 | < 0.01 |
| | NS | – | – | – | – | < 0.01 | < 0.01 |

Chole = Cholecystectomy; Inf = inflammatory; NS = not significant. B = benign; Inf = Iinflammatory; – not applicable. Each group has three values of *P* (or NS when *P* < 0.05): Top = baseline (resting), middle = after 6 h incubation without endotoxin (unstimulated cells) and bottom = after 6 h incubation with endotoxin (stimulated cells).

### RESULTS

### Assessment of monocyte tissue factor activity in breast and colorectal cancer

The median and IQR of mTF activity for breast and colorectal diseases are shown in Figures 1 and 2 and statistical comparisons detailed in Table 2.

In the breast disease groups (Figure 1), there was no significant difference between the normal and the other two control groups (hernia or cholecystectomy, and benign non-inflammatory breast disease). Inflammatory benign disease showed an elevation in mTF levels compared with each control group for the stimulated cells only. Although the numbers in the inflammatory group were small, the statistical test gave $P < 0.01$. On the other hand, the malignant groups showed variable significant increases over the control groups for the baseline, unstimulated and the stimulated cells (Table 2). There was no significant difference between the benign inflammatory group and the malignant group for all the three parameters studied.

For colorectal disease (Figure 2), the three control groups displayed no significant differences when tested against each other. The inflammatory benign disease group showed significantly raised mTF over all controls for baseline ($P < 0.05$), unstimulated ($P < 0.05$) and stimulated cells ($P < 0.01$). Malignancy conferred a significant increase over each control group for all mTF parameters measured; baseline ($P < 0.05$), unstimulated ($P < 0.05$) and stimulated cells ($P < 0.01$). Table 2 includes all relevant *P*-values. No significant difference was observed between benign inflammatory and malignant patients.

mTF in patients with breast malignancy gave results above the upper quartile range of the normal controls for resting 46.6%, unstimulated 32.8% and stimulated cells 61.9%, while patients with colorectal cancer showed 33.9% for resting, 33.3% unstimulated and 66.7% stimulated cells. The stimulated mTF activity of the colorectal group showed the highest increase compared with controls or patients with breast malignancy (Figure 3).

### Tumour grade or stage

Tumour grading was carried out according to histological criteria. There is an increase in mTF corresponding to a higher tumour grade or stage. This trend did not reach statistical significance for resting, unstimulated or LPS-stimulated cells (Kruskal–Wallis).

### Patient survival

There was a trend towards decreased mTF activity in longer surviving patients with malignancy. Again, the differences were not statistically significant (Mann–Whitney).

### DISCUSSION

Using a two-stage KCA for mTF measurements, patients with malignant tumours showed significant increases in mTF levels when compared with the controls or the relevant benign non-inflammatory conditions. Similarly, patients with inflammatory conditions (mastitis, mammary duct ectasia, ulcerative colitis and diverticulitis) showed significantly increased mTF levels when compared with controls or benign non-inflammatory disease groups. No significant differences were observed between the appropriate benign inflammatory and malignant disease groups. However, the numbers of inflammatory breast disease accrued were small and should not be over-interpreted. These results agree with those reported by Carvalho (1995) and for urinary TF activity by Lwaleed et al (1999).

Inflammatory conditions are known to enhance mTF expression (Hogg, 1983) and isolated monocytes from patients with Crohn's disease were noticed to express elevated levels of mTF (Edwards et al, 1987). When the benign groups of the present study were sub-divided into organ-specific non-inflammatory and inflammatory diseases, the former group showed comparable levels with the controls. Patients undergoing hernia repair and cholecystectomy who had a normal ESR also showed a low variation in mTF levels equivalent to that of the normal control groups. Similar results

 *© Cancer Research Campaign 1999*

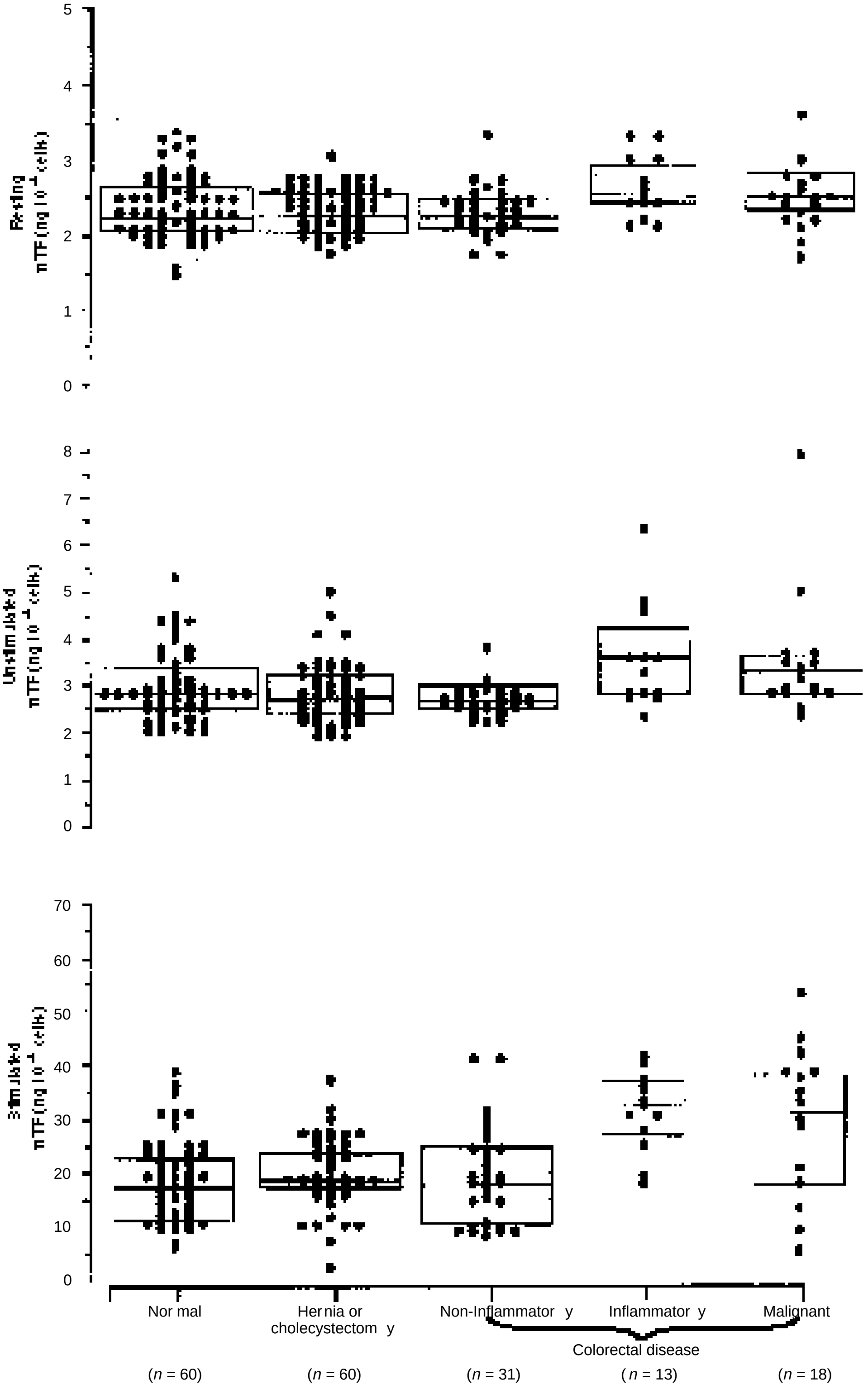

**Figure 2** Monocyte tissue factor activity (ng $10^{-6}$ cells) in normal controls, patients undergoing hernia repair or cholecystectomy, non-inflammatory and inflammatory benign disease, and malignant disease of the colorectum, for baseline (resting), after 6 h incubation without endotoxin (unstimulated cells) and after 6 h incubation with endotoxin (stimulated cells)

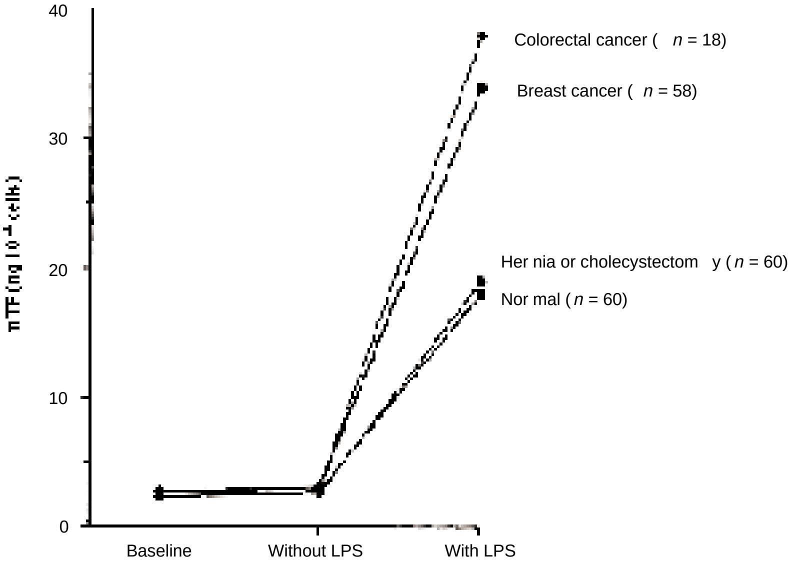

**Figure 3** Increase in monocyte tissue factor activity (ng $10^{-6}$ cells) after lipopolysaccharide stimulation in normal controls, patients undergoing hernia repair or cholecystectomy, and patients with breast and colorectal cancer have been reported (Dasmahapatra et al, 1987; Carvalho, 1995) and these get further support by immunohistochemical and in situ detection studies (Contrino et al, 1996). It appears that TF assays respond with equal sensitivity to the benign inflammatory and malignant conditions and the link might be immunologically mediated (Edwards et al, 1987; Hogg, 1983; Østerud et al, 1990).

Although inflammatory and malignant conditions showed increased levels of mTF as compared with the controls, there was an overlap between the groups. This may have been exacerbated by factors such as the heterogeneity of the various groups, including the stage of evolution of the disease or treatment schedules. All patients on anticoagulant and/or steroids were excluded from the study. However, of the patients with Crohn's disease treated with prednisolone inadvertently studied and retrospectively excluded, few showed normal mTF levels.

A comparison of the three levels of mTF activity measured (baseline, unstimulated and stimulated) shows that the latter discriminates better between the groups studied, consistent with the previous reports (Dasmahapatra et al, 1987; Carvalho 1995). Despite the fact that stimulated mTF levels gave the best results this perhaps reflects not only the in vivo condition, but possibly changes in the cellular phospholipid compositions caused by LPS in vitro. TF activity is known to depend greatly on the composition of complexed phospholipid (Nemerson and Gentry, 1986). In that respect, baseline mTF activity may be more physiologically relevant. Unlike the stimulated levels, baselines are closely associated with the antigenic levels measured by flow cytometry (Carvalho, 1995). However, both levels may be required in assessing the PCA of mTF in disease states (Francis et al, 1995).

A previous study showed no relationship between tumour grade or stage and mTF levels (Carvalho, 1995) and it was postulated that elevated mTF levels are present from the initial growth of the primary tumour. However, the incidence of clotting abnormalities rises as the disease progresses (Naschitz, 1996) and the present study demonstrated an increase in the mTF levels associated with tumour grade or stage. This is particularly true for the colorectal group where patients with Dukes' B and C had higher mTF levels than Dukes' A.

Although mTF levels increased in the tumours studied, a greater increase was observed for colorectal cancer as compared to breast tumour. While it is known that colorectal cancer expresses elevated levels of cellular TF (Szczepanski, 1988) and that higher levels of free TF were observed for colorectal tissues compared to breast tissues (El-Baruni, 1990), there is no obvious reason to explain this phenomenon. Inflammation is also known to significantly enhance mTF production and colorectal cancer is often associated with ulceration and bacterial infection which is known to stimulate mTF expression (Østerud and Flaegstad, 1983). Another important factor which may have contributed to this finding is that most colon cancer patients present at a later stage of disease progression and the tumours are physically larger, particularly in relation to breast cancer which is subject to screening.

There was a trend towards decreased mTF activity in longer surviving patients with malignancy, as reported previously (Carvalho, 1995). These results are also consistent with the finding that mTF activity increased with tumour grade or stage.

In conclusion, LPS enhances mTF activity and stimulated cells can better distinguish patients with cancer from normal controls and benign diseases in the absence of inflammation. Thus LPS-stimulated mTF levels may play a role in screening patients that are at risk and most likely to benefit from further investigation for cancer. mTF levels were raised in patients with higher tumour grade or stage. This suggests a role for mTF levels in assessing tumour progression and response to treatment.

### REFERENCES

Auger MJ and Mackie MJ (1987) Monocyte procoagulant activity in breast cancer. *Thromb Res* **47**: 77–84

Bach RR, Nemrson Y and Konigsberg WK (1981) Purification and characterization of bovine tissue factor. *J Biol Chem* **256**: 8324–8331

 *© Cancer Research Campaign 1999*

Carson SD and Konisberg WH (1980) Cadmium increases tissue factor (coagulation factor III) activity by facilitating its reassociation with lipids. *Science* **208**: 307–309

Carvalho MG (1995) Monocyte tissue factor in malignancy. PhD thesis. Southampton University.

Contrino J, Hair G, Kreutzer D and Rickles FR (1996) In situ detection of expression of tissue factor in vascular endothelial cells: correlation with the malignant phenotype of human breast tissue. *Nature Med* **2**: 209–215

Dasmahapatra KS, Cheung NK, Spillert C and Lazaro E (1987) An assessment of monocytes procoagulant activity in patients with solid tumours. *J Surg Res* **43**: 158–163

Edwards RL, Rickles FR and Cronlund M (1981) Abnormalities of blood coagulation in patients with cancer. Mononuclear cell tissue factor generation. *J Lab Clin Med* **98**: 917–928

Edwards RL, Levine JB, Green R, Duffy M, Mathews E, Brande W and Rickles FR (1987) Activation of blood coagulation in Crohn's disease: Increased fibrinopeptide A levels and enhanced generation of monocyte tissue factor activity. *Gastroenterology* **92**: 329–337

El-Baruni (1990) Factor X-activating procoagulant in normal and malignant breast tumour. PhD thesis. Southampton University.

Francis JL, Carvalho M and Francis DA (1995) The clinical value of tissue factor assays. *Blood Coag Fibrinol* **6**: 37–44

Guha A, Bach RR, Konigsberg W and Nemerson Y (1986) Affinity purification of human tissue factor. Interaction of factor VII and tissue factor in detergents micelles. *Proc Natl Acad Sci USA* **83**: 299–302

Hogg N (1983) Human monocytes are associated with the formation of fibrin. *J Exp Med* **157**: 473–485

Lorenzet R, Peri G, Locati D, Allavena P, Colucci M, Semeraro N, Mantovanti A and Donati MB (1983) Generation of procoagulant activity by mononuclear phagocytes: a possible mechanism contributing to blood clotting activation within malignant tissues. *Blood* **62**: 271–273

Lwaleed BA, Chisholm M and Francis JL (1997) Elevated blood monocyte tissue factor levels in patients with cancer (Abstract). *Br J Cancer* **75**: 45

Lwaleed BA, Chisholm M and Francis JL (1999) Urinary tissue factor levels in patients with breast and colorectal cancer. *J Pathol* **187**: 291–294

Mellor H, Taylor I, Roath S and Francis JL (1989) Whole blood procoagulant activity in breast and colorectal cancer. *J Clin Pathol* **42**: 489–494

Morgan D, Edwards RL and Rickles FR (1988) Monocyte procoagulant activity as a peripheral marker of clotting activation in cancer patients. *Haemostasis* **18**: 55–65

Muller AD, van Dam-Mieras MCE and Hemker HC (1985) Measurement of macrophage cellular procoagulant activity. *Haemostasis* **15**: 108–113

Naschitz JE, Yeshurun D, Eldar S and Lev LM (1996) Diagnosis of cancer-associated vascular disorders. *Cancer* **77**: 1759–1767

Nemerson Y (1992). The tissue factor pathway of blood coagulation. *Semin Haemost* **29**: 170–176

Nemerson Y and Bach R (1982) Tissue factor revisited. *Prog Hemost Thromb* **6**: 237–261

Nemerson Y and Gentry R (1986) An ordered addition, essential activation of model of the tissue factor pathway of coagulation: evidence for conformational change. *Biochemistry* **25**: 4020–4033

Østerud B and Bjorkild E (1982) The production and availability of tissue thromboplastin in cellular populations of whole blood exposed to various concentration of endotoxin: an assay for detection of endotoxin. *Scand J Haematol* **29**: 175–184

Østerud B and Due Jr J (1984) Blood coagulation in patients with benign and malignant tumours before and after surgery. Special reference to thromboplastin generation in monocytes. *Scand J Haematol* **32**: 258–264

Østerud B and Flaegstad T (1983) Increased thromboplastin activity in monocytes of patients with meningococcal infection: related to an unfavourable prognosis. *Thromb Haemost* **49**: 5–7

Østerud B, Olsen JO and Wilsgard L (1990) The role of arachidonic acid release and lipoxygenase pathway in lipopolysaccharide-induced thromboplastin activity in monocytes. *Blood Coagul Fibrinol* **1**: 41–46

Parmar J, Taylor I, Roath OS and Francis JL (1990) Procoagulant activity in whole blood from patients with breast and colorectal cancer. *Blood Coag Fibrinol* **1**: 127–132

Szczepanski M, Bardadin K, Zawadzki J and Pypno W (1988) Procoagulant activity of gastric, colorectal, and renal-cancer is factor-VII-dependent. *J Cancer Res Clin Oncol* **114**: 519–522

*© Cancer Research Campaign 1999*
